# High fluoroquinolone resistance proportions among multidrug-resistant tuberculosis driven by dominant L2 *Mycobacterium tuberculosis* clones in the Mumbai Metropolitan Region

**DOI:** 10.1186/s13073-022-01076-0

**Published:** 2022-08-22

**Authors:** Viola Dreyer, Ayan Mandal, Prachi Dev, Matthias Merker, Ivan Barilar, Christian Utpatel, Kayzad Nilgiriwala, Camilla Rodrigues, Derrick W. Crook, the CRyPTIC Consortium, Derrick W. Crook, Timothy E. A. Peto, A. Sarah Walker, Sarah J. Hoosdally, Ana L. Gibertoni Cruz, Joshua Carter, Sarah Earle, Samaneh Kouchaki, Yang Yang, Timothy M. Walker, Philip W. Fowler, Daniel Wilson, David A. Clifton, Zamin Iqbal, Martin Hunt, Jeff Knaggs, Daniela M. Cirillo, Emanuele Borroni, Simone Battaglia, Arash Ghodousi, Andrea Spitaleri, Andrea Cabibbe, Sabira Tahseen, Kayzad Nilgiriwala, Sanchi Shah, Camilla Rodrigues, Priti Kambli, Utkarsha Surve, Rukhsar Khot, Stefan Niemann, Thomas Kohl, Matthias Merker, Harald Hoffmann, Katharina Todt, Sara Plesnik, Nazir Ismail, Shaheed Vally Omar, Lavania Joseph Dumisani Ngcamu, Nana Okozi, Shen Yuan Yao, Guy Thwaites, Thuong Nguyen Thuy Thuong, Nhung Hoang Ngoc, Vijay Srinivasan, David Moore, Jorge Coronel, Walter Solano, George F. Gao, Guangxue He, Yanlin Zhao, Aijing Ma, Chunfa Liu, Baoli Zhu, Ian Laurenson, Pauline Claxton, Robert J. Wilkinson, Anastasia Koch, Ajit Lalvani, James Posey, Jennifer Gardy, Jim Werngren, Nicholas Paton, Ruwen Jou, Mei-Hua Wu, Yu-Xin Xiao, Lucilaine Ferrazoli, Rosangela Siqueira de Oliveira, James Millard, Rob Warren, Annelies Van Rie, Simon Grandjean Lapierre, Marie-Sylvianne Rabodoarivelo, Niaina Rakotosamimanana, Camus Nimmo, Kimberlee Musser, Vincent Escuyer, Ted Cohen, Jean-Philippe Rasigade, Thierry Wirth, Nerges Mistry, Stefan Niemann

**Affiliations:** 1 Molecular and Experimental Mycobacteriology, Research Center Borstel, Borstel, Germany; 2 German Center for Infection Research (DZIF), Partner Site Hamburg-Lübeck-Borstel-Riems, Borstel, Germany; 3 The Foundation for Medical Research, Mumbai, India; 4 Evolution of the Resistome, Research Center Borstel, Borstel, Germany; 5 CRyPTIC Consortium, Oxford, UK; 6 Department of Microbiology, P. D. Hinduja National Hospital and Medical Research Centre, Mumbai, India; 7 Nuffield Department of Medicine, University of Oxford, Oxford, UK; 8 Centre International de Recherche en Infectiologie, INSERM U1111, CNRS UMR5308, ENS Lyon, University of Lyon, Lyon, France; 9 Institut Des Agents Infectieux, Hospices Civils de Lyon, Lyon, France; 10 Institut de Systématique, Evolution, Biodiversité, UMR-CNRS 7205, Muséum National d’Histoire Naturelle, Université Pierre Et Marie Curie, Université Des Antilles, Ecole Pratique Des Hautes Etudes, Sorbonne Universités, Paris, France; 11 EPHE, PSL University, Paris, France

**Keywords:** Tuberculosis, Resistant TB, Multidrug-resistant TB, Fluoroquinolone resistance, India; Pre-XDR/XDR-TB, Pre-XDR/XDR-TB transmission, Transmission success

## Abstract

**Background:**

Multidrug-resistant (MDR) *Mycobacterium tuberculosis complex* (MTBC) strains are a serious health problem in India, also contributing to one-fourth of the global MDR tuberculosis (TB) burden. About 36% of the MDR MTBC strains are reported fluoroquinolone (FQ) resistant leading to high pre-extensively drug-resistant (pre-XDR) and XDR-TB (further resistance against bedaquiline and/or linezolid) rates. Still, factors driving the MDR/pre-XDR epidemic in India are not well defined.

**Methods:**

In a retrospective study, we analyzed 1852 consecutive MTBC strains obtained from patients from a tertiary care hospital laboratory in Mumbai by whole genome sequencing (WGS). Univariate and multivariate statistics was used to investigate factors associated with pre-XDR. Core genome multi locus sequence typing, time scaled haplotypic density (THD) method and homoplasy analysis were used to analyze epidemiological success, and positive selection in different strain groups, respectively.

**Results:**

In total, 1016 MTBC strains were MDR, out of which 703 (69.2%) were pre-XDR and 45 (4.4%) were XDR. Cluster rates were high among MDR (57.8%) and pre-XDR/XDR (79%) strains with three dominant L2 (Beijing) strain clusters (Cl 1–3) representing half of the pre-XDR and 40% of the XDR-TB cases. L2 strains were associated with pre-XDR/XDR-TB (*P* < 0.001) and, particularly Cl 1–3 strains, had high first-line and FQ resistance rates (81.6–90.6%). Epidemic success analysis using THD showed that L2 strains outperformed L1, L3, and L4 strains in short- and long-term time scales. More importantly, L2 MDR and MDR + strains had higher THD success indices than their not-MDR counterparts. Overall, compensatory mutation rates were highest in L2 strains and positive selection was detected in genes of L2 strains associated with drug tolerance (*prpB* and *ppsA*) and virulence (*Rv2828c*). Compensatory mutations in L2 strains were associated with a threefold increase of THD indices, suggesting improved transmissibility.

**Conclusions:**

Our data indicate a drastic increase of FQ resistance, as well as emerging bedaquiline resistance which endangers the success of newly endorsed MDR-TB treatment regimens. Rapid changes in treatment and control strategies are required to contain transmission of highly successful pre-XDR L2 strains in the Mumbai Metropolitan region but presumably also India-wide.

**Supplementary Information:**

The online version contains supplementary material available at 10.1186/s13073-022-01076-0.

## Background

Multidrug-resistant (MDR) tuberculosis (TB) caused by *Mycobacterium tuberculosis* complex (MTBC) strains resistant to at least isoniazid (INH) and rifampicin (RMP) poses a great challenge to global TB control. More than 400,000 new MDR-TB cases are notified annually [1]; 50% of these coming from India (27%), China (14%), and countries of the Russian Federation (9%). This makes them the epi-centers of the current MDR-TB epidemic and key countries for the implementation of successful future intervention against MDR-TB [1]. In India, about 36% of the MDR-TB cases are reported to have additional resistance to fluoroquinolones (FQ) [1, 2] and around 3% of MDR-TB cases are estimated to be extensively drug resistant (XDR, World Health Organization [WHO] classification until April 2021, i.e., additional resistance to one of the fluoroquinolones as well as to one of the injectable drugs). No data is available based on the new WHO definitions for pre-XDR (MDR with additional resistance against a FQ) and XDR (pre-XDR with additional resistance to one of the WHO Group A drugs) [3–5].

The treatment of MDR-TB patients is longer, based on less effective, more toxic drugs, and in 2019, the cure rate was only 57% on a global level [1, 4]. With 39%, the treatment success rate is even lower for XDR-TB patients [3]. As ineffective treatment is an important factor driving transmission [6], the potential of MDR/XDR MTBC strains to transmit may be even higher compared to susceptible MTBC strains [7].

Considering the MDR-TB epidemiology in India, a better understanding of drug resistance development, in particular resistance to FQs and new MDR-TB drugs such as bedaquiline (BDQ), and MTBC transmission success in the region is crucial [8, 9]. Indeed, it is of particular importance to understand the origins and driving forces of the MDR-TB epidemic in the country including ongoing transmission of already highly resistant clones [10–12], but only few studies have used state-of-the-art whole genome sequencing (WGS) combined with epidemiological techniques to investigate transmission in India so far [13–16].

To address these knowledge gaps, we performed a retrospective genomic epidemiological analysis based on WGS data of 1852 MTBC strains mainly from the Mumbai metropolitan region, India. The strains were obtained from a tertiary care hospital laboratory in Mumbai that provides comprehensive drug susceptibility testing (DST) of MTBC strains. WGS data were used to determine MTBC lineage, resistance to first- and second-line drugs, and transmission inference of MTBC strains based on allele and single-nucleotide polymorphism (SNP) differences. Furthermore, to disentangle the influences of genetic background, drug resistance, and compensatory mutations on the transmission success of MTBC strains, we used the time scaled haplotypic density (THD) method [17, 18]. This method uses genetic distances to assign a relative index of epidemic success to each strain in a population over a specified time scale, allowing in turn correlating success with other strain characteristics [19]. The relative success of lineages was compared over a long-term timescale of 200 years and a short-term timescale of 20 years, as used in previous studies of MTBC [20].

## Methods

### Study design

**Fig. 1:**
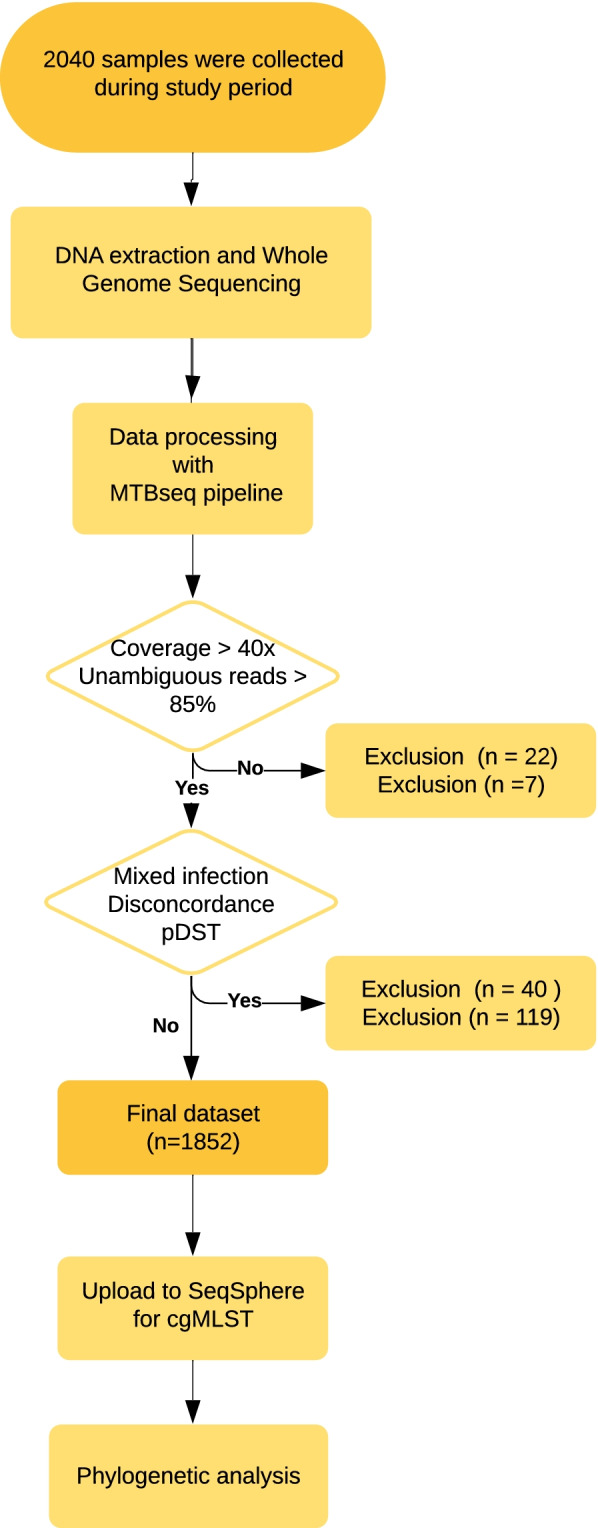
Study flowchart. In- and exclusion criteria for strains are reported in the two rhombuses. Final dataset consists of 1852 strains

A total of 2040 MTBC strains from patients (one isolate per patient) were retrospectively collected for the CRyPTIC Consortium Project between February 2017 and May 2018 (15 months) from the laboratory of a tertiary care hospital in India. CRyPTIC stands for “Comprehensive Resistance Prediction for Tuberculosis: an International Consortium” and is a worldwide collaboration between TB research institutions all over the world to achieve better, faster, and more targeted treatment of MDR-TB via genetic resistance prediction. Sequential culture positive samples referred by private physicians to the hospital laboratory for further investigation were included in the study. Given that the Xpert® MTB/RIF (Cepheid, USA) test, a test for detection of both the presence of the MTBC genome in patient specimen and the presence of genomic sequences of the main mutations responsible for rifampicin resistance, is being done at peripheral centers, there is a potential bias toward RMP-resistant samples in the study collection, which was intended as the CRyPTIC study aimed at defining drug resistance mechanisms. Considering that in Mumbai around 5000 MDR-TB cases are reported annually, the study covered approx. 16% of MDR cases occurring in the region (*n* = 1016/6250) (source: https://portal.mcgm.gov.in/) [21]. Datasets of 1852 strains (90.8%) were included in the final analysis, while 188 datasets were excluded due to less than 40 × coverage (*n* = 22), proportion of unambiguous reads were below 85% (*n* = 7), mixed infections with two MTBC strains (49) and major discrepancies between the assessment of resistance categories (i.e., susceptible, RMP resistant, MDR, pre-XDR, and XDR, *n* = 119, Additional file 1: Table S1) based on genotypic resistance determinates and minimum inhibitory concentrations in microtiter plates (Fig. 1). Of the 1852 MTBC strains included in the final analysis, 1773 were collected from the Mumbai Metropolitan region, and 46 from distal parts of Maharashtra and neighboring States/Union Territories and 33 from a hospital in Himachal Pradesh, North India. Approval for the CRyPTIC study was obtained from the Health Ministry’s Screening Committee (HMSC), Government of India dated 6 October 2016, the Institutional Ethics Committee (IEC) of The Foundation for Medical Research, Mumbai (Ref nos. FMR/IEC/TB/01a/2015 and FMR/IEC/TB/01b/2015), and Institutional Review Board of P.D. Hinduja Hospital and Medical Research Centre, Mumbai (Ref no. 915–15-CR [MRC]).

### Molecular methods

Genomic DNA was isolated from the 2040 patient samples using FastPrep24 lysis method (MP Biomedicals, California, USA) as per standard protocol and quantified using Qubit (Life Technologies, Carlsbad, California, USA). Libraries for WGS were prepared using Nextera XT DNA Library Prep Kit, and sequencing was performed on the Illumina NextSeq500 machine as per the manufacturer’s protocol (Illumina Inc., San Diego, California, USA) producing 2 × 151 base pair reads.

### Genome analysis

All WGS data were analyzed using the MTBseq pipeline (Version 1.0.3) [22]. The reads were mapped to the reference sequence *M. tuberculosis* H37Rv (GenBank ID: NC_000962.3) with the Burrows-Wheeler Aligner (BWA) [23]. Initial mapping was refined using tools from the Genome Analysis Toolkit (GATK) [24] and SAMtools [25] to, e.g. exclude PCR artifacts, correct alignment errors around small insertions/deletions (InDels) and recalibrate base quality scores. Minimum criteria for variants (SNPs and InDels) were set to four reads coverage per direction (forward and reverse) and a variant frequency of 20% for resistance prediction and 75% for phylogenetic analysis, respectively. Phylogenetic lineages (MTBC lineages and known Beijing subgroups) were inferred from specific SNPs based on Coll *et al.* [26] and Merker *et al.* [10].

### Genome-based resistance prediction and cluster analysis

Polymorphisms in 27 drug resistance-associated genes that are involved in drug resistance mechanisms and three compensatory target genes (*rpoA*, *rpoC*, compensate fitness effects of *rpoB* mutations in RMP-resistant strains [27], and *ahpC* upstream region, compensate fitness effects of catalase [*katG*] deficit in INH-resistant strains [28]) were analyzed (Additional file 2: Table S2). Primary cluster analysis was done using the core genome multi locus sequence typing (cgMLST) method as described previously [29]. A minimum spanning tree was calculated by ignoring pairwise missing values and using a cluster alert with a 12 alleles distance to cover a timespan of the last 25 years referring to Meehan *et al.* [30]. SNP-based phylogenies were calculated as described previously [31]. Concatenated SNP alignment was used to calculate a maximum likelihood (ML) phylogeny using IQ-TREE software [32] with ModelFinder option and ascertainment bias correction. We employed ultrafast bootstrap (UFBoot) approximation with 1000 replicates combined with a further optimizing step to reduce the risk of overestimating the branch support. Phylogenetic trees were mid-point rooted using FigTree v1.4.4 and annotated using the online tool EvolView [33].

### Geospatial analysis

Geospatial mapping of collected samples was done using available fuzzy locations and pin codes. Nearest Neighbor Index was computed based on the average distance from each feature to its nearest neighboring feature. The Cluster and Outlier Analysis was used to identify spatial clusters if any based on Anselin Local Morans [34]. Geospatial information is included in Additional file 3: Table S3.

### Homoplasy analysis

Homoplasy, i.e., the occurrence of identical SNPs in phylogenetically unrelated isolates, was detected with HomoplasyFinder (https://github.com/JosephCrispell/homoplasyFinder) as described earlier [35] and using R (version 4.0.3). Based on a concatenated SNP alignment, we calculated ML-trees of all L2 strains and from strains of clusters 1–3, respectively. For the actual SNP alignment used in the homoplasy analysis, we re-introduced SNPs in genes associated with drug resistance and bacterial fitness.

### Epidemic success analysis

The THD success index was computed as described elsewhere [18] using R package THD (https://github.com/rasigadelab/thd) based on the matrix of genetic distances between isolates (SNP counts). User-defined parameters were a mutation rate of 10^−7^ mutation per site per year, an effective genome size (number of positions retained for SNP calling) of 4 × 10^6^ and time scales of 20 years and 200 years as indicated in the text. Differences of THD distribution across groups were tested using a two-sided Mann–Whitney *U* test. In line with the exploratory nature of the analysis, no correction for multiple hypothesis testing was performed. We grouped resistance categories as follows: not-MDR, MDR and MDR + , where not-MDR included S, RMP-resistant (RR) and nonMDR strains, MDR included all MDR-only strains and in MDR + all strains with resistance category pre-XDR or higher were grouped together.

### Statistics

Descriptive statistics was performed for patients’ demographics as well as for lineages, resistance categories and clustering status of MTBC strains. Data derived from genomic analysis of clinical isolates were analyzed statistically using IBM SPSS Statistics Software for Windows (version 19) and R (version 3.6.1). For univariate analysis of potential factors associated with pre-XDR/XDR-TB, we performed a Fisher’s exact test. Factors with a significant result in the univariate model were included into a multivariate logistic regression analysis. Odds ratios with 95% confidence interval (CI) were estimated and variables with *P*-values less than 0.05 were taken as significant characteristics.

## Results

### Study population

The final dataset consisted of 1852 strains (90.8%), while 188 datasets were excluded due to several reasons described in the methods and Fig. 1.

Of the 1852 patients, 55.5% (*n* = 1027) were female, and 44.5% (*n* = 824) were male. TB cases were mostly diagnosed within the age group of 18 to 40 years (*n* = 1081; 58.4%) and were widely distributed in Mumbai and its suburban region (Additional File 4: Fig. S1, Additional file 5: Table S4). The mean age of the whole population was 33.7 years (SD 16.3). All data are summarized in Additional file 3: Table S3.

### Drug resistance and MTBC population structure

**Fig. 2:**
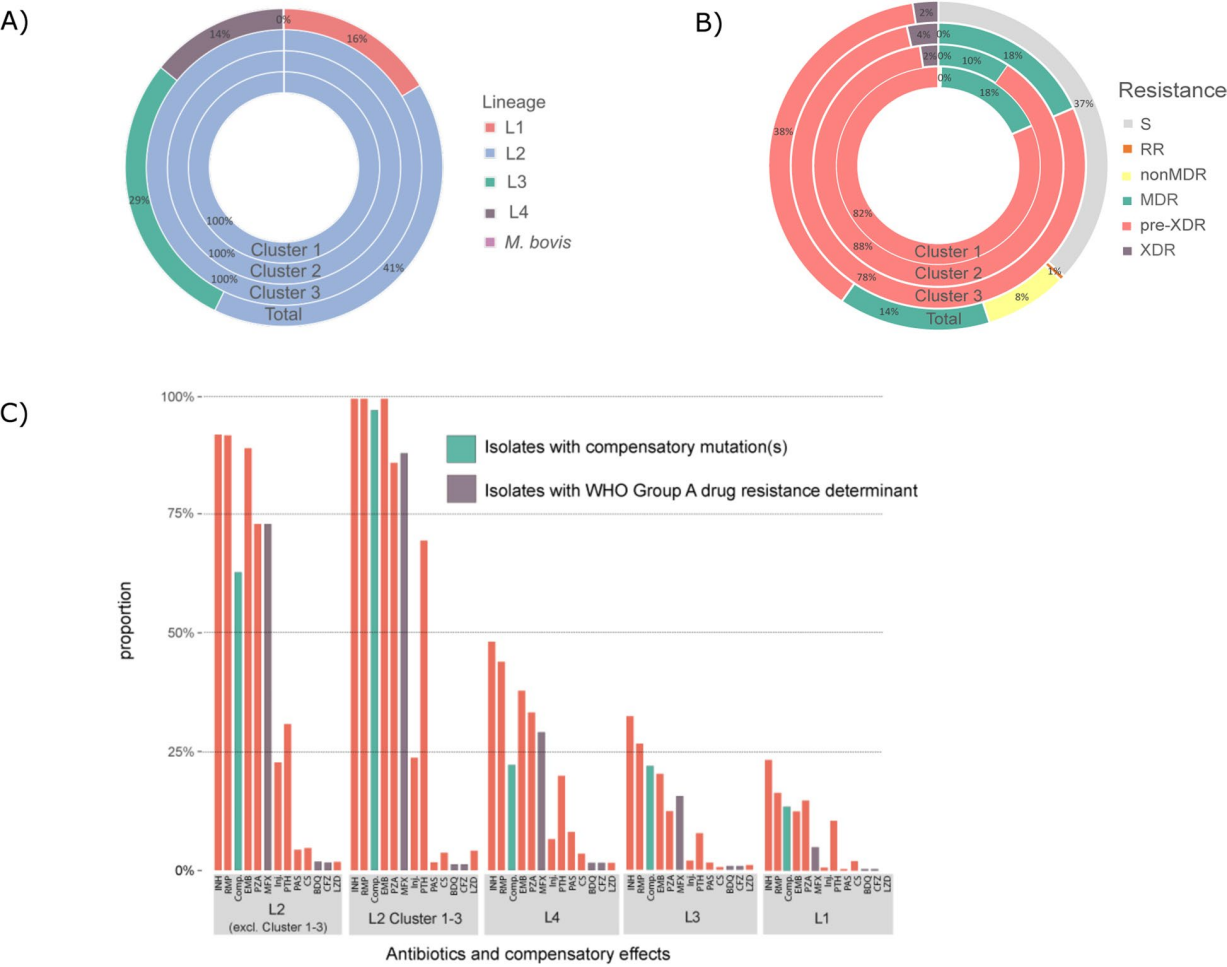
Genotype and resistance category distribution of strains across 1852 clinical isolates (Total) and within the three major clusters. **A** Distribution of resistance category across the 1852 isolates; resistance categories are RR (rifampicin resistant), nonMDR (resistant, but not multi drug resistant [MDR]), MDR, pre-XDR (pre-extensively drug resistant), and XDR (extensively drug resistant); of the total strain population, about 37% are susceptible (S) to all drugs, 38% of strains are pre-XDR whereas 2.4% are XDR in the cohort. The resistant category distribution of clusters 1, 2, and 3 differs, with all strains being at least MDR). **B** Proportion of strains with known resistance mutations per lineage. Each bar represents a specific antibiotic or compensatory effect. L2 strains, especially the ones from clusters 1–3, have the highest proportion of resistance mutations and also compensatory effects. Abbreviations: INH, isoniazid; RMP, rifampicin; Comp., compensatory mutation; EMB, ethambutol; PZA, pyrazinamide; MFX, moxifloxacin; Inj., injectables; PTH, prothionamide; PAS, para-aminosalycilic acid; CS, cycloserine; BDQ, bedaquiline; CFZ, clofazimine; LZD, linezolid; L1, Lineage 1; L2, Lineage 2; L3, Lineage 3; L4, Lineage 4

The 1852 MTBC strains were then classified in MDR, pre-XDR and XDR according to the new WHO definitions [5]. In total, 1016 strains were at least MDR (54.9%) and 836 (45.1%) were not-MDR, including 681 (36.8%) pan-susceptible strains and 154 strains (8.3%) with variable resistances other than MDR (Fig. 2, Additional file 5: Table S4). Among the 1016 MDR strains, 703 strains were further classified as pre-XDR due to additional FQ resistance (38% of the total population, and 69.2% of the MDR MTBC strains) and 45 were XDR (2.4% of the total population, and 4.4% of the MDR MTBC strains, Fig. 2, Additional file 3: Table S3, Additional file 5: Table S4). Twenty-one strains had mutations in the genes *Rv0678* and *atpE* which mediate resistance against BDQ, of which eleven also had another mutation in *rplC* or *rrl* conferring resistance to linezolid (LZD, Additional file 3: Table S3). Overall, 38 strains had mutations in these genes and thus are resistant against linezolid.

Lineage 2 strains (L2, Beijing/East Asia) constituted 41% of the total collection (*n* = 756), followed by Lineage 3 (L3, Delhi/CAS, *n* = 531, 29%), Lineage 1 (L1, East Africa Indian, EAI, *n* = 303, 16%), and Lineage 4 (L4, Euro-American, *n* = 260, 14%) strains (Fig. 2). L2 strains are overrepresented in drug resistance strains, especially in those harboring multiple drug resistances, while strains of the other three lineages show an opposite trend (Fig. 2c, Additional file 4: Fig. S2, Additional file 5: Table S4). Indeed, 80% of all pre-XDR and 66.7% of all XDR strains belong to L2 (Additional file 5: Table S4). The overall number of resistance mutations and drug resistance rates of first-line and WHO group A, B, and C MDR-TB treatment drugs are clearly higher in L2 strains (Fig. 2c, Additional file 4: Fig. S2).

### Genome-based cluster analysis

A cgMLST-based cluster analysis employing a threshold of a maximum distance of 12 alleles grouped 801 (43%) of the 1852 strains into 96 clusters, ranging in size from two to 258 strains (Additional file 3: Table S3, Additional file 5: Fig. S4). Clusters were identified among all four major MTBC lineages; however, the three largest clusters (clusters 1–3) comprised only L2 strains (Fig. 2, Additional file 5: Table S4). Cluster 1 comprised 258 strains of the previously defined L2 subgroup “Asian/Africa 2”, cluster 2 comprised 127 strains classified as “Ancestral 3”, and 87 strains in cluster 3 could not be further classified [10] (Additional file 3: Table S3).

Stratified to the different resistance categories and considering the entire collection, the cluster rate was 7% in not-MDR, 57.8% in MDR, 79.1% in pre-XDR, and 77.8% in XDR strains (Additional file 5: Table S4). Strains of the three dominant L2 clusters (CL 1–3) represent half of the pre-XDR and 40% of the XDR-TB cases. Just cluster 1 strains accounted for 28% of the pre-XDR and 28.9% of the XDR MTBC strains.

Notably, all L2 strains and particularly strains of the three largest clusters evolved high resistance rates to first-line antibiotics (Fig. 2c). Cluster 1–3 strains were almost exclusively resistant to INH, RMP, ethambutol (EMB), and streptomycin (SM, Fig. 2c, Additional file 4: Fig. S4). L2 strains also developed high FQ resistance rates (81.6–90.6%) rendering them at least pre-XDR (see below), with a FQ resistance rate between 83 and 91% in cluster 1–3 strains (Fig. 2c, Additional file 4: Fig. S4). Resistance to other WHO group A drugs was present in 5% in cluster 1 strains, and 13 out of the 258 cluster 1 strains (5%) were already classified as XDR based on the new WHO classification (Fig. 2, Additional file 5: Table S4).

In addition to resistance mutations, we identified the presence of putative compensatory mutations across all four major lineages in the genes *rpoA*, *rpoB*, and *rpoC* which were not related to resistance per se and co-occurred with canonical rifampicin resistance determining mutations. Likewise, to the high rifampicin resistance proportions among L2 strains (244/284, 85.9%), proportions of compensatory mutations were high in L2 strains (174/284, 61.3%) and within clusters 1–3 nearly all strains (455/472, 96.4%) had at least one compensatory mutation (Fig. 2c).

### Factors associated with pre-XDR/XDR-TB

In the univariate statistical analysis, no associations among sex and age groups with pre-XDR/XDR-TB were identified (Table 1). L2 strains (*P* < 0.001), belonging to a cluster (*P* < 0.001), and belonging to clusters 1–3 (*P* < 0.001; *P* < 0.01; *P* = 0.09) increased the odds of pre-XDR/XDR. Infections with L1, L3, and L4 strains had lower odds to be classified as pre-XDR/XDR compared to L2 strains (Table 1).

**Table 1:** Characteristics of MDR and pre-XDR/XDR cases with factors associated with pre-XDR/XDR-TB based on univariate and multivariate analysis

	**MDR**		**Pre-XDR/XDR**		**Univariate analysis**				**Multivariate logistic regression**			
	*n*	%	*n*	%	OR	95% lower	95% upper	*P*-value	Adjusted OR	95% lower	95% upper	Adjusted *P*-value
Total	268	100%	748	100%								
Gender												
M	114	43%	320	43%	1.00	0.75	1.35	1				
F	153	57%	428	57%								
Unknown	1	0%	0	0%								
Age												
< 18	37	14%	93	12%	0.89	0.58	1.38	0.59				
18–40	168	63%	489	65%	1.12	0.83	1.52	0.46				
40–60	45	17%	138	18%	1.12	0.77	1.66	0.58				
> 60	17	6%	29	4%	0.6	0.31	1.18	0.12				
Unknown	1	0%	0	0%								
Lineage												
Lineage 1 (EAI)	37	14%	13	2%	0.11	0.05	0.22	< 0.001	0.19	0.09	0.41	< 0.001
Lineage 2 (Beijing)	121	45%	592	79%	4.60	3.38	6.28	< 0.001	2.09	1.26	3.45	< 0.01
Lineage 3 (Delhi-CAS)	70	26%	69	9%	0.29	0.20	0.42	< 0.001	0.54	0.32	0.89	0.02
Lineage 4 (Euro-American)	40	15%	74	10%	0.63	0.41	0.97	0.03				
*M. bovis*	0	0%	0	0%								
Clustering												
Yes	155	58%	591	79%	2.74	2.01	3.74	< 0.001	0.92	0.62	1.35	0.66
No	113	42%	157	21%								
Cluster												
Cluster 1	44	16%	213	28%	2.03	1.40	2.98	< 0.001	1.19	0.74	1.91	0.46
Cluster 2	12	4%	115	15%	3.87	2.09	7.85	< 0.001	2.36	1.19	4.69	0.01
Cluster 3	16	6%	71	9%	1.65	0.93	3.10	0.09	1.09	0.58	2.08	0.79

In the multivariate analysis, the odds of a strain being pre-XDR/XDR was twice as high for strains belonging to L2 (adjusted odds ratio [aOR] 2.09, 95% CI 1.26, 3.45) and for strains belonging to cluster 2 (aOR 2.36, 95% CI 1.19, 4.69), 50% lower for strains belonging to L3 (aOR 0.54, 95% CI 0.32, 0.89) and 80% lower for strains belonging to L1 (aOR 0.19, 95% CI 0.09, 0.41; Table 1 and Additional file 6: Table S5) as compared to L4.

### High-resolution SNP-based analysis of cluster 1–3 MTBC strains

To get in-depth view on the transmission dynamics and evolution of the most dominant strains in our collection, we performed a high-resolution SNP-based analysis on the L2 strains of allele clusters 1–3.

**Fig. 3:**
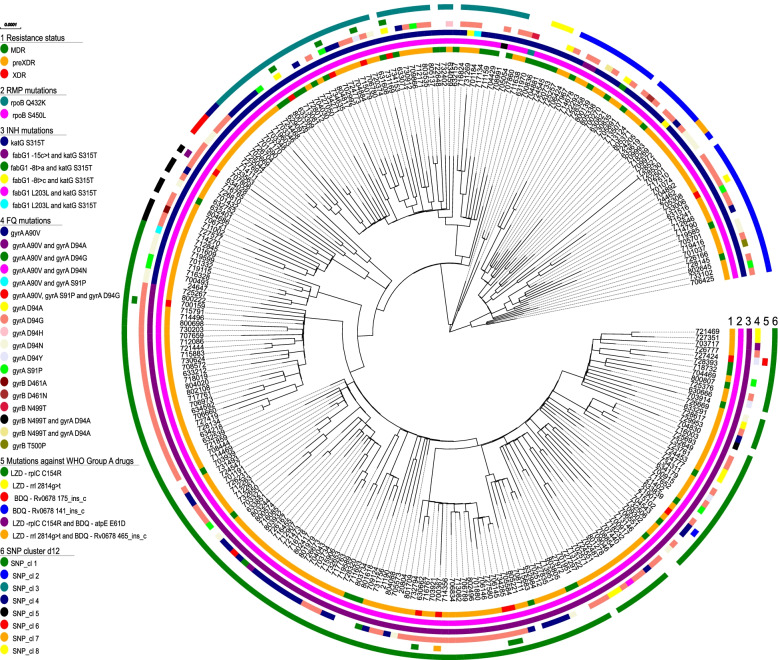
Maximum likelihood phylogeny based on the concatenated SNP sequence of 258 MTBC strains from allele-based cluster 1. The concatenated SNP sequence consists of 356 parsimony-informative, 961 singleton sites, and 1106 constant sites; mutations related to respective drugs and resistance status are color coded and expressed as annotation rings on the tree. SNP-based clusters with maximum distance of 12 (d12) is plotted on the outer ring. Abbreviations: INH, isoniazid; EMB, ethambutol; PZA, pyrazinamide; FQ, fluoroquinolones

Using a maximum SNP distance of 12, a total of 239 cluster 1 strains could be grouped into eight SNP-based clusters (SNP_cl), ranging in size from two to 143 (Fig. 3, Additional file 3: Table S3). The largest SNP cluster is SNP_cl 1 with 143 strains, followed by SNP_cl 2 and SNP_cl 3, which comprise 37 and 41 strains, respectively. The phylogeny of the strains in maximum likelihood phylogeny based on the concatenated SNP sequence (356 parsimony-informative, 961 singleton sites, 1106 constant sites) is in line with particular resistance types, and, thus, confirmed the clonality of the strains in particular sub-branches, e.g., by carrying the same *rpoB*, *embB*, or *pncA* mutations (Fig. 3).

The close relationship and likely transmission of pre-XDR and XDR strains was further confirmed by the grouping of strains into several closely related subgroups in the phylogeny that share the same FQ resistance mutations, e.g., *gyrA* A90V, or even double mutations such as *gyrA* A90V and *gyrA* D94G (Fig. 3, Additional file 3: Table S3), pointing toward a common ancestor that acquired these mutations before the clone started spreading as pre-XDR/XDR clone in the Mumbai area.

**Fig. 4:**
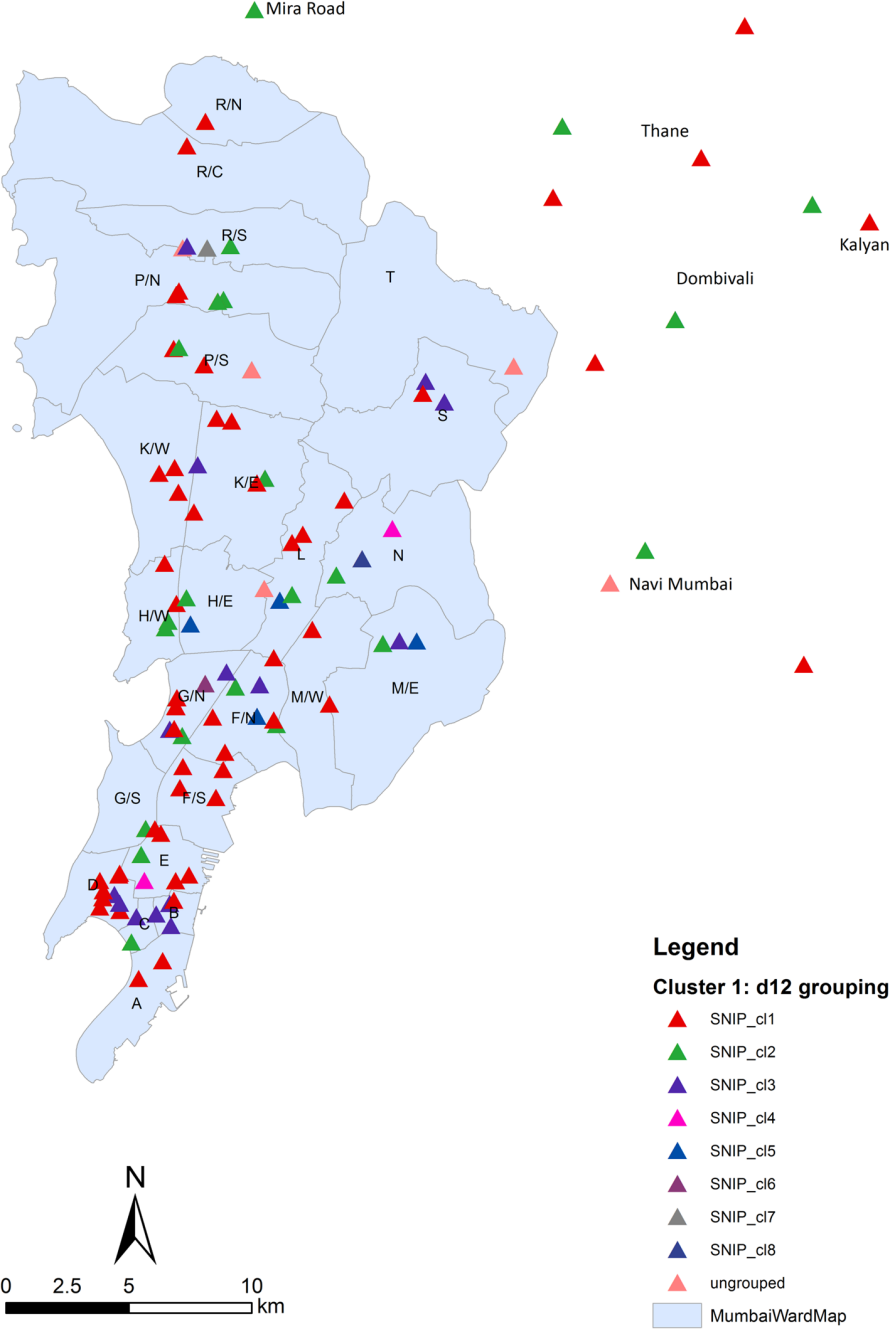
Geographical occurrence of patients with maximum SNPs distance of 12 for cluster 1 across Mumbai Metropolitan Region. The geographical distribution underlines the widespread of all cluster 1 SNP subgroups across the city and its neighboring areas. Boundaries of the map for the neighboring regions are not available online

To test if strains of particular subgroups were spreading within Mumbai, we linked the SNP clusters with geographical occurrence (Fig. 4, Additional file 3: Table S3). However, this analysis revealed that strains of all cluster 1 SNP subgroups occur in all parts of the study region indicating a wide distribution of these clones in the study area. Finally, we screened the NGS datasets for compensatory mutations that have been previously described to enhance the transmissibility of MDR strains [10, 11, 27]. This analysis revealed that 243 of the 258 cluster 1 strains carry at least one compensatory mutation in *rpoC* (Additional file 3: Table S3).

SNP-based analysis of strains from clusters 2 and 3 confirmed their high clonality with virtually all strains belonging to 1 SNP group (Additional file 4: Fig. S5). The phylogeny of cluster 2 and 3 strains reveals a similar pattern than observed for cluster 1 strains. Subgroups share same patterns of resistance mutations indicating their acquisition by a common ancestor followed by clonal transmission (Additional file 4: Fig. S5). This also applies for pre-XDR and/or XDR MTBC strains.

### Epidemicity, strain success, and genomic/antibiotic resistance backgrounds

**Fig. 5:**
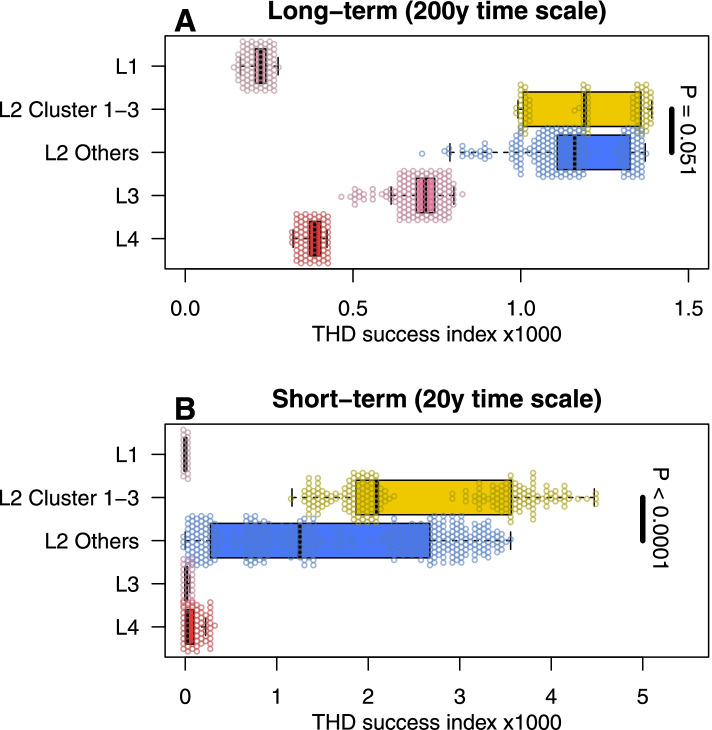
MTBC lineage 2 exhibits greater epidemic success than Lineages 1, 3, and 4. Shown are boxplots and distribution of THD success indices in MTBC Lineages 1–4, with Lineage 2 split into clusters 1–3 and other strains. THD success indices were larger in Lineage 2 compared with other lineages, using both a long-term (**A**) and a short-term (**B**) analysis time scale. Within Lineage 2, clusters 1–3 had similar long-term success as other strains over a long-term time scale (**A**) but had superior success in the short-term (**B**), suggesting that strains from clusters 1–3 became successful only recently. *P*-values obtained from 2-sided Mann–Whitney *U* test

**Fig. 6:**
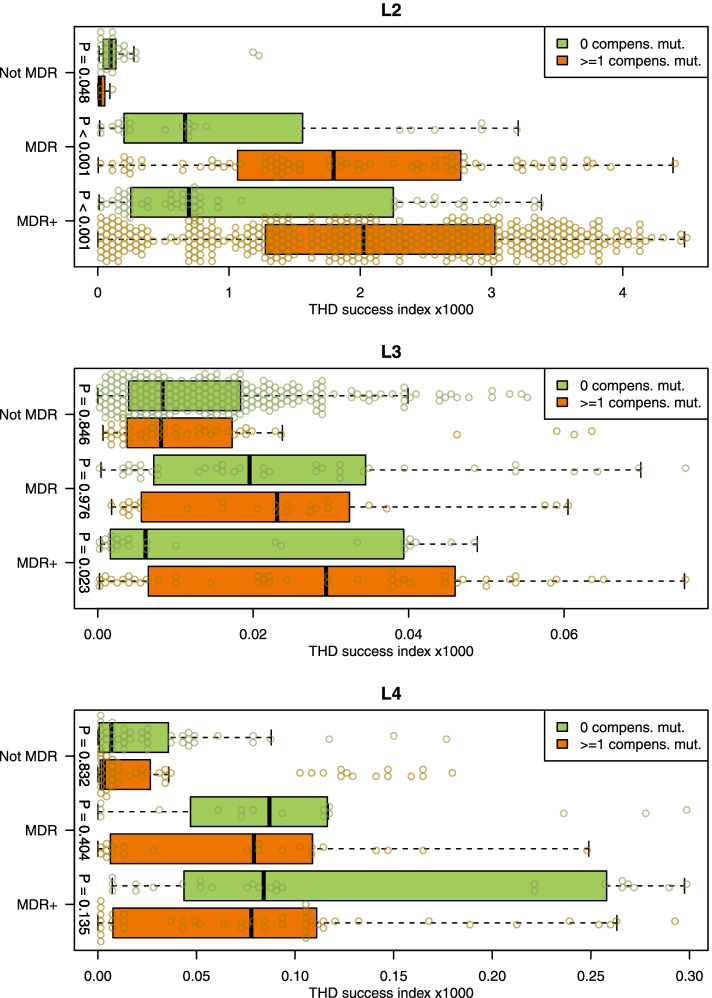
Compensatory mutations correlate with increased epidemic success of MDR MTBC strains. Shown are boxplots and distribution of THD success indices with a 20-year time scale in MTBC lineages 2–4 with resistance profile categorized as not-MDR, MDR, and MDR + (pre-XDR/XDR). Within each lineage and resistance profile, strains without compensatory mutations (green) were compared with those with at least 1 compensatory mutation (orange). Compensatory mutation(s) correlated with increased success indices in MDR lineage 2, MDR + lineages 2 and 3, but not in lineage 4 strains. In nonMDR lineage 2 strains, compensatory mutation(s) correlated with lesser success. *P*-values obtained from 2-sided Mann–Whitney *U* test. Lineage 1 was excluded and clusters 1–3 were pooled with other lineage 2 isolates because all lineage 1 and clusters 1–3 strains had at least 1 compensatory mutation

In a first step, we compared THD success indices across the main lineages present in this study (L1 to L4), as well as across the identified clusters. Interestingly, strains of L2 lineage outperformed the three other lineages in terms of epidemic success, both in the short- and long-term time scales (Fig. 5 and Additional file 7: Table S6). When considering L2 alone, the strains belonging to clusters 1–3 displayed higher THD indices (2.1, interquartile range [IQR] 1.9 to 3.56) than the other L2 strains (1.3, IQR 0.28 to 2.67) using a short-term, 20-year time scale (*P* < 0.0001). This pattern did not hold for the 200-year time scale, suggesting a recent emergence and expansion of clusters 1–3. We evaluated the impact of the strains’ antibiotic profile, and presence of compensatory mutations on the pathogen epidemic success. For L4, MDR and MDR + strains had larger THD success compared to the not-MDR strains; however, the presence of compensatory mutations was not associated with increased THD indices (Fig. 6 and Additional file 8: Table S7). For L3, the pattern was somehow similar, though MDR + strains harboring compensatory mutations performed substantially better than the MDR + strains lacking those mutations (*P* = 0.023). The situation was dramatically different for L2. First, the THD index was much higher (nearly two orders of magnitude) than in other lineages; but more importantly MDR and MDR + strains harboring compensatory mutations performed nearly three times better than the strains lacking such compensatory mutations (0.66, IQR 0.20 to 1.56 versus 1.8, IQR 1.07 to 2.75; and 0.7, IQR 0.25 to 2.25 versus 2.02, IQR 1.28 to 3.02).

### Genomic success factors of L2 MTBC strains

In order to identify genetic factors that contribute to the success of L2 MTBC strains in general and cluster 1–3 strains in particular, we performed a homoplasy analysis. Specifically, we screened the phylogeny for mutations that occur independently in the phylogenetic tree, i.e., mutations which are not explained by a common ancestry, and which are potentially under positive selection [35]. Within the L2 dataset, we identified 172 mutations under positive selection, thereof 84 mutations in genes associated with drug resistance and compensatory mechanisms (Additional file 9: Table S8). Thirty-one out of 84 resistance and compensatory mutations possibly under selection in the L2 dataset are also identified in the cluster 1–3 datasets, suggesting ongoing resistance evolution and adaptation of strains of the three major clusters (Additional file 9: Table S8). Among L2 strains in general, we identified signatures for positive selection in the gene *prpR*, which was recently associated with conditional drug tolerance [36], and *ppsA*, which has been shown to be upregulated in rifampicin-resistant strains [37]. In addition, we pointed out mutations in four genes encoding human epitope regions and mutations in the gene *Rv2828c.* The latter has been reported to be associated with a more widespread radiological pathology, in particular the mutation *Rv2828c* T141R [38]. Here, we found homoplasy and evolutionary convergence among L2 with 609/756 isolates harboring the combination *Rv2828c* T141R + S128C, and one isolate with *Rv2828c* T141K + S128C (Additional file 9: Table S8).

To further support this hypothesis, we also calculated the THD success indices for strains with a mutation in *Rv2828c* and without (Additional file 4: Fig. S6). On a long-term evolutionary time scale (200 years), the THD index was higher in any L2 isolate harboring *Rv2828c* mutations (Additional file 4: Fig. S6 A). However, this increased epidemic success was only confirmed for L2 cluster 1–3 isolates on a short-term (20 years) analysis time scale (Additional file 4: Fig. S6 B).

## Discussion

We performed a large-scale genome-based study analyzing 1852 MTBC strains mainly from the Mumbai Metropolitan region, to define determinants of the MDR, pre-XDR, and XDR epidemic in one of the highest populated metropolitan areas of the world. Our data show very high rates of pre-XDR strains among the MDR strain population in this study; 69.2% of all MDR strains were pre-XDR and 4.4% were XDR, using the new WHO classification. The remarkable shift toward pre-XDR is mediated by high frequencies of FQ resistance mutations that, in combination with particular mutations against WHO group A drugs, result in XDR-TB (WHO classification since April 2021) [5]. As FQ resistance is reported as main determinant of MDR-TB treatment failure [39–41], such high FQ resistance rates potentially severely affect the efficacy of new MDR-TB treatment regimens. Further, we identified MTBC strains belonging to L2, and particularly three main clusters, as main drivers of the pre-XDR epidemic in the Mumbai Metropolitan Region. Strains of the three dominant MDR clones have very high first-line, FQ resistance rates of more than 80% and already acquired additional drug resistances, including resistance to BDQ and LZD. THD analysis confirmed the high epidemic success of L2 strains in the region and a high capacity to spread as MDR, pre-XDR, and XDR variants with a tangible effect of compensatory mutations in L2 strains only.

The overall high rate of FQ-resistant pre-XDR among all MDR strains that is, with more than 80%, even higher in strains of CL 1–3, is particularly concerning as this is rendering one of the most effective drugs of the short and long MDR-TB regimen non-effective [42]. The predominant mutations in the FQ-resistant strains from our study were *gyrA* D94G (41%) and *gyrA* A90V (21%; Additional file 3: Table S3), the former mutation contributing to high level resistance [43]. To the best of our knowledge, a comparable shift of resistance from MDR toward pre-XDR has not been reported from other regions in the world, though it has been evident in Mumbai over the last two decades [9, 44, 45]. Previous reports based on the old WHO definition estimate that approximately 6% of the MDR-TB patients have an XDR-TB [3], though the rate was reported lower for India [1, 2]. In our study, we observe a higher XDR-TB rate of 17.9% using the old WHO definition (data not shown) and already a 4.4% XDR-TB rate using the new definition. This shows relevant resistance rates to other WHO Group A drugs such as BDQ and LZD that, in line with the high FQ resistance rate, argue for thorough control of the use of BDQ containing regimens in the region, especially as the main driver of BDQ resistance are mutations in *Rv0678* also mediating resistance to clofazimine [46–48].

A further striking finding of our study is the strong association of L2 strains with pre-XDR/XDR-TB linked to a very high genomic cluster rate (85%) of L2 strains. Strains of the three major L2 clusters account for more than half of the pre-XDR and 42.2% of all XDR-TB cases, and just cluster 1 strains account for approx. 30% of the pre-XDR/XDR MTBC strains. This demonstrates that L2 strains are important drivers of the MDR/pre-XDR-TB epidemic which is in line with an increasing proportion of L2/Beijing strains in the Mumbai Metropolitan region considering data gathered over the last two decades [9, 44, 49]. Our data also underline the potential of MDR MTBC strains to evolve high numbers of drug resistances and yet to efficiently transmit, even when they become pre-XDR and potentially XDR. The clonal expansion of particular pre-XDR/XDR clones with combined resistance to all first-line drugs and FQs reduces available group A, B, and C drugs proposed for the treatment of MDR-TB cases to a minimal set, and renders the use of the short MDR-TB regimen for patients infected with such strains impossible [3, 42]. Geographical mapping showed that strains of the dominant cluster 1 are dispersed across all districts of the Mumbai metropolitan area of more than 28 million inhabitants. Indeed, our THD analysis underlined the high epidemicity of L2 stains compared to other strains of other lineages especially when MDR, pre-XDR, and XDR strains where compared with not-MDR (S, RR, and nonMDR) strains. Obviously, the drug-resistant L2 strains are transmitted more efficiently with a pronounced impact of compensatory mutations that was only visible in drug-resistant L2 strains in our study. As indicated by growth competition experiments in a previous study [50], L2 MDR/pre-XDR strains appear to have a high competitive fitness leading to transmission success. In our study, this feature is now accompanied with high resistance rates and additional acquisition of compensatory mutations. Further, an association between BCG vaccine escape and efficient spread of Beijing strains has also been proposed [51, 52]. In addition to high rates of previously described compensatory mutations, e.g., in *rpoC*, we found further evidence for mutation under positive selection that lead to drug tolerance, e.g., *prpB* [36] and *ppsA* [37] and increased virulence mediated by *Rv2828c* mutations in codon 141 and 128, which were associated with an increased lung pathology, previously [38] and potentially allow for an increased spread of L2 strains in India. Also, THD analyses pointed toward an increased long-term (200 years) epidemiological success for L2 strains harboring mutations in *Rv2828c*. On a short-term time scale (20 years), we only found an increased THD index for L2 cluster 1–3 strains. The difference between short- and long-term success might be further influenced by elevated drug resistance proportions among L2 cluster 1–3 strains, and a moderate short-term impact of *Rv2828c* mutations on the transmission success.

Our study has limitations. We only investigated MTBC strains from the laboratory of one tertiary care hospital in Mumbai which represents community samples from a pool of private physicians from the Mumbai Metropolitan Region. Although our study covered approx. 16% of MDR cases occurring in the region, it may not be representative for the whole Mumbai metropolitan area and for the rest of India. Still, our geographical mapping shows that the study captured cases from the main districts of Mumbai as well as from other areas in India. However, considering our findings, larger investigations on the MDR/pre-XDR/XDR proportions and on the spread of dominant pre-XDR/XDR MTBC strains in Mumbai and other parts of India urgently need to be performed.

## Conclusions

In conclusion, our data indicate that the pre-XDR/XDR-TB epidemic can be propelled by few clones with high proportions of pre-existing drug resistances and ongoing selection for compensatory mutations, virulence determinants, and resistances against new drugs and drugs of last resort. This trend is confirmed by the L2 THD index, which is nearly two orders of magnitude higher than for the other lineages, indicating that those strains have some constitutive evolutionary advantages compared to other MTBC lineages, as already described earlier [10, 53, 54]. Even more worrying is the fact that MDR and MDR + L2 strains are performing much better compared to not-MDR strains and that, in our dataset, acquisition of compensatory mutations was accompanied by a threefold increase of their THD success index, challenging therefore the classical view of high fitness costs in MDR + strains [55, 56]. Thus, successful control of the DR epidemic in Mumbai urgently requires measures for stopping the transmission of MDR/pre-XDR/XDR L2 strains. As pre-XDR strains are FQ resistant, treatment options are limited and rapid adaptation of treatment strategies, for example, comprehensive resistance detection for better design of personalized effective treatment regimens, need to be established. It is likely that the uninformed use of treatment regimens including the newest MDR-TB drugs without precise knowledge of individual resistance patterns and close patient monitoring will result in further resistance development as described already [47, 57–59] and ongoing transmission of even more resistant strains. BDQ or LZD resistance has already emerged in relevant proportions in the dominant clones; thus, the future development needs to be monitored, e.g., using prospective molecular surveillance studies, and the effect of FQ resistance in combination with high backbone resistance levels on the effect of BDQ containing regimens needs to be closely monitored. The extent of the spread of the dominant pre-XDR/XDR clones in India needs to be urgently considered.

## Supplementary Information

**Additional file 1: Table S1.** Phenotypic drug susceptibility (DST) and correlation with genotypic DST.

**Additional file 2: Table S2.** Genes analyzed.

**Additional file 3: Table S3.** Data table.

**Additional file 4: Figure S1.** Geographical distribution of the strains investigated. Strains were plotted on a map according to the geographical position of the submitting center and color coded by the resistance category. All categories came from the whole study region. **Figure S2.** Boxplot of the amount of resistance mutations per lineage. Strains belonging to Lineage 2 (L2) show more resistance mutations compared to the other lineages. Especially, the strains belonging to the allele cluster C1-C3 have the largest amount of resistance mutations. Center line, median; box limits, upper and lower quartiles; whiskers, 1.5x interquartile range; points, outliers. **Figure S3.** Minimum spanning tree based on the analysis of 2891 alleles of the core genome of the 1852 *M. tuberculosis* complex strains investigated. Missing values were ignored for pairwise comparisons. Strains are color-coded by the respective lineage name. EAI and EAI Manila (Lineage 1), Beijing (Lineage 2), Delhi- CAS (Lineage 3) and Euro- American, H37Rv-like, Haarlem, LAM, mainly T, S-type, Ural and X-type (Lineage 4). Allele clusters are highlighted by color-shaded branches. **Figure S4.** Barplot of resistance profiles of the strains belonging to allele cluster 1, 2 and 3. Resistance profiles in %. A) Resistance profile of allele Cluster 1, B. Resistance profile of allele Cluster 2, C. Resistance profile of allele Cluster 3. **Figure S5.** Maximum likelihood (ML) phylogeny of strains belonging to cluster 2 and 3. Mutations related to respective drugs and resistance status are color coded on the annotation rings of the tree. **Figure S6.** Rv2828c mutations correlate with increased epidemic success among MTBC lineage 2 isolates. Shown are boxplots and distribution of THD success indices in MTBC lineages 2 belonging or not to clusters 1-3. Among clusters 1-3 isolates, THD success indices were larger in isolates harboring Rv2828c mutations using both a long-term (A) and a short-term (B) analysis time scale. Among other L2 isolates, THD success indices were large in Rv2828c-positive isolates using a long-term timescale but not a short-term time-scale. This suggest that Rv2828c mutations were beneficial among L2 isolates in the long term, but only beneficial to cluster 1-3 isolates in the short term. P-values obtained from 2-sided Mann-Whitney U-test.

**Additional file 5: Table S4.** Characteristics of analyzed samples divided by resistance group.

**Additional file 6: Table S5.** Multivariate logistic regression results.

**Additional file 7: Table S6.** THD success indices in MTBC lineages.

**Additional file 8: Table S7.** THD success indices in MTBC lineages with and without compensatory mutations.

**Additional file 9: Table S8.** Homoplasy analysis.

**Additional file 10: Table S9.** ENA accession numbers.

## Data Availability

Fastq files are available at the European Nucleotide Archive under accession ID PRJEB41116 (https://www.ebi.ac.uk/ena/browser/view/PRJEB41116?show=reads) [60]. Further details on the accession numbers of specific datasets can be found in Additional file 10: Table S9. All other data generated or analyzed during this study are included in this article and its additional information files.

## Abbreviations

aOR: Adjusted odds ratio
BDQ: Bedaquiline
BWA: Burrows-Wheeler Aligner
cgMLST: Core genome multi locus sequence typing
CI: Confidence interval
CL: Cluster
DST: Drug susceptibility testing
EMB: Ethambutol
ENA: European Nucleotide Archive
FQ: Fluoroquinolones
GATK: Genome Analysis Toolkit
HMSC: Health Ministry’s Screening Committee
IEC: Institutional Ethics Committee
InDels: Insertions/deletions
INH: Isoniazid
IQR: Interquartile range
L1: Lineage 1
L2: Lineage 2
L3: Lineage 3
L4: Lineage 4
LZD: Linezolid
MDR: Multidrug resistant
ML: Maximum likelihood
MTBC: *Mycobacterium tuberculosis* Complex
RMP: Rifampicin
RR: Rifampicin resistant
SM: Streptomycin
SNP: Single-nucleotide polymorphism
SNP_cl: SNP-based clusters
TB: Tuberculosis
THD: Time scaled haplotypic density
UFBoot: Ultrafast bootstrap
WGS: Whole genome sequencing
WHO: World Health Organization
XDR: Extensively drug resistant
