# Supplementary material for: High fluoroquinolone resistance proportions among multidrug-resistant tuberculosis driven by dominant L2 Mycobacterium tuberculosis clones in the Mumbai Metropolitan Region
Source: Genome Med. 2022 Aug 22;14:95. doi: 10.1186/s13073-022-01076-0 (PMC9394022; doi:10.1186/s13073-022-01076-0)
Supplement: Supplementary file 4 — Additional file 4: Figure S1. Geographical distribution of the strains investigated. Strains were plotted on a map according to the geographical position of the submitting center and color coded by the resistance category. All categories came from the whole study region. Figure S2. Boxplot of the amount of resistance mutations per lineage. Strains belonging to Lineage 2 (L2) show more resistance mutations compared to the other lineages. Especially, the strains belonging to the allele cluster C1-C3 have the largest amount of resistance mutations. Center line, median; box limits, upper and lower quartiles; whiskers, 1.5x interquartile range; points, outliers. Figure S3. Minimum spanning tree based on the analysis of 2891 alleles of the core genome of the 1852 M. tuberculosis complex strains investigated. Missing values were ignored for pairwise comparisons. Strains are color-coded by the respective lineage name. EAI and EAI Manila (Lineage 1), Beijing (Lineage 2), Delhi- CAS (Lineage 3) and Euro- American, H37Rv-like, Haarlem, LAM, mainly T, S-type, Ural and X-type (Lineage 4). Allele clusters are highlighted by color-shaded branches. Figure S4. Barplot of resistance profiles of the strains belonging to allele cluster 1, 2 and 3. Resistance profiles in %. A) Resistance profile of allele Cluster 1, B. Resistance profile of allele Cluster 2, C. Resistance profile of allele Cluster 3. Figure S5. Maximum likelihood (ML) phylogeny of strains belonging to cluster 2 and 3. Mutations related to respective drugs and resistance status are color coded on the annotation rings of the tree. Figure S6. Rv2828c mutations correlate with increased epidemic success among MTBC lineage 2 isolates. Shown are boxplots and distribution of THD success indices in MTBC lineages 2 belonging or not to clusters 1-3. Among clusters 1-3 isolates, THD success indices were larger in isolates harboring Rv2828c mutations using both a long-term (A) and a short-term (B) analysis time scale. Among other L2 [file 13073_2022_1076_MOESM4_ESM.docx]

Supplementary Figures


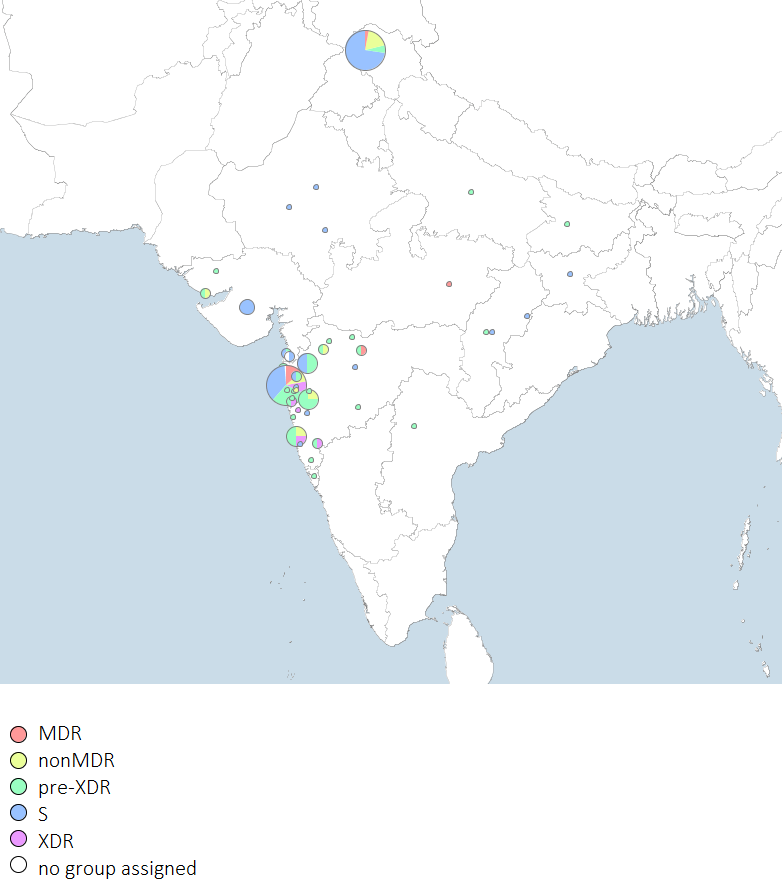
**Fig S1**. Geographical distribution of the strains investigated. Strains were plotted on a map according to the geographical position of the submitting center and color coded by the resistance category. All categories came from the whole study region.

Abbreviations: S- Susceptible to all drugs, nonMDR – resistant but not multi-drug resistant, MDR – multi-drug resistant, pre-XDR – pre-extensively drug resistant, XDR – extensively drug resistant


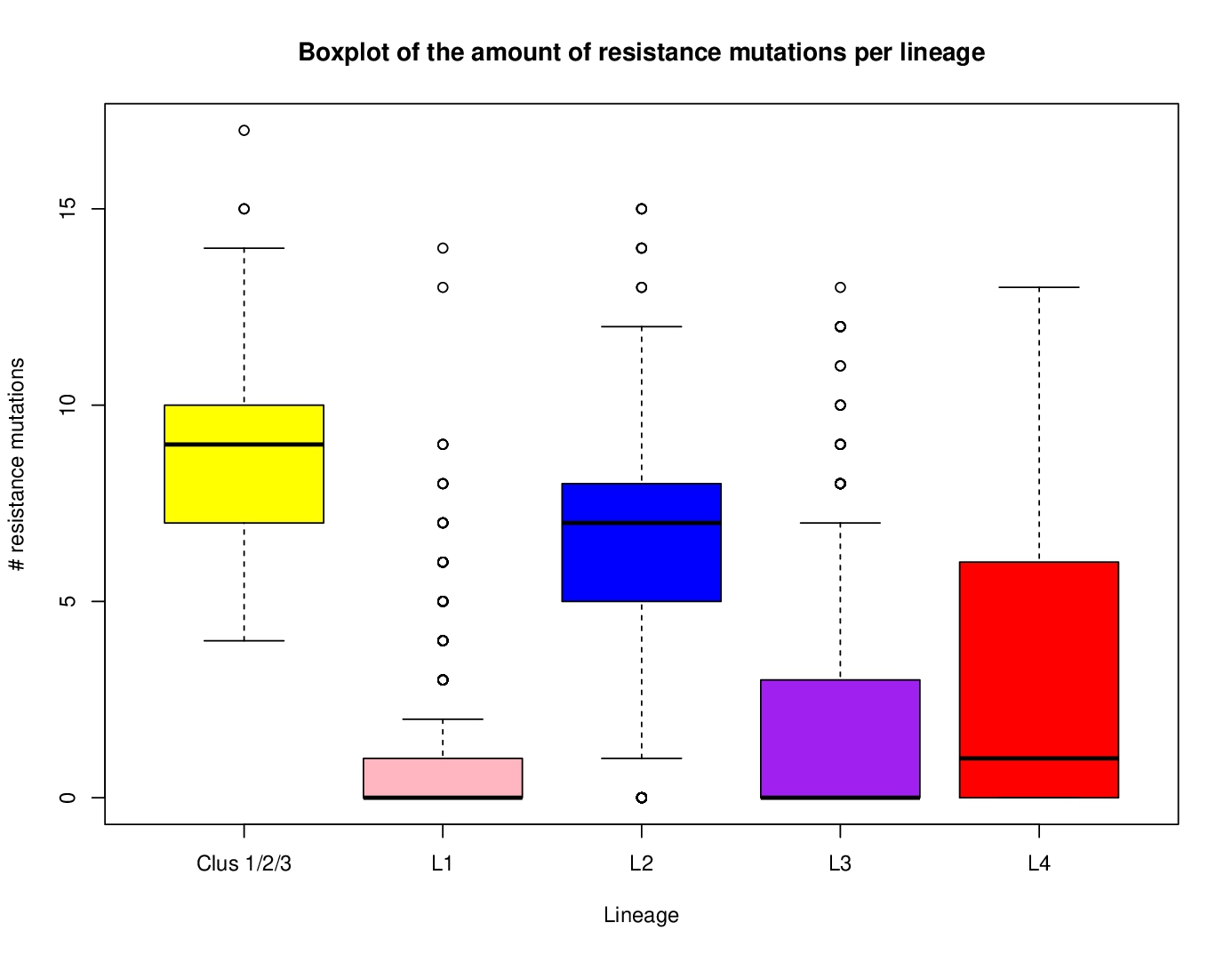
**Fig S2**. Boxplot of the amount of resistance mutations per lineage. Strains belonging to Lineage 2 (L2) show more resistance mutations compared to the other lineages. Especially, the strains belonging to the allele cluster C1-C3 have the largest amount of resistance mutations. Center line, median; box limits, upper and lower quartiles; whiskers, 1.5x interquartile range; points, outliers.


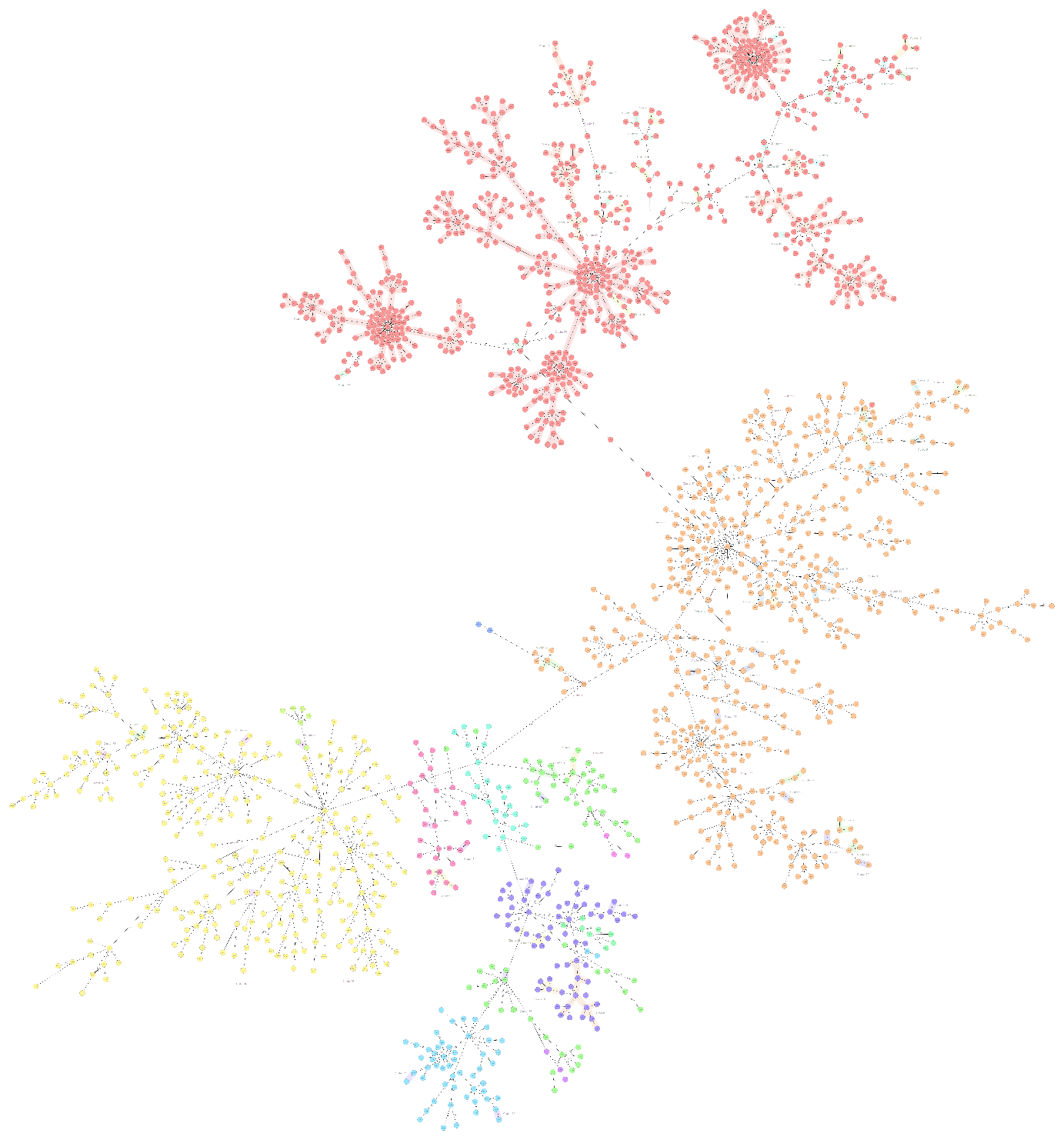

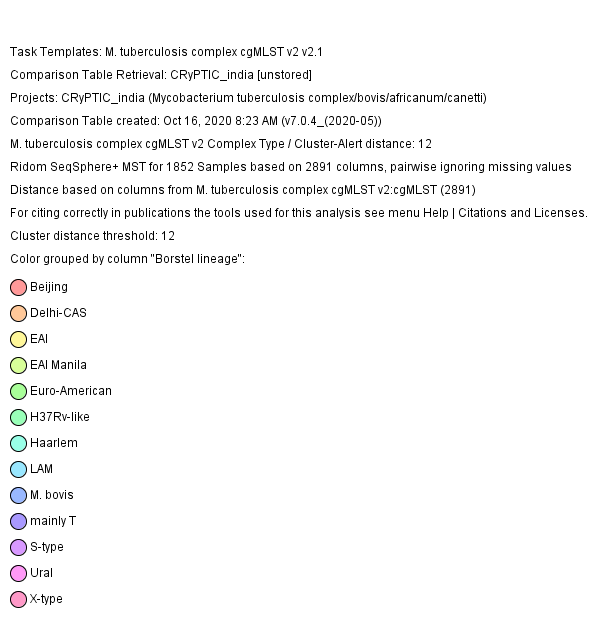


**Fig S3**. Minimum spanning tree based on the analysis of 2 891 alleles of the core genome of the 1 852 *M. tuberculosis* complex strains investigated. Missing values were ignored for pairwise comparisons. Strains are color-coded by the respective lineage name. EAI and EAI Manila (Lineage 1), Beijing (Lineage 2), Delhi- CAS (Lineage 3) and Euro- American, H37Rv-like, Haarlem, LAM, mainly T, S-type, Ural and X-type (Lineage 4). Allele clusters are highlighted by color-shaded branches.

A


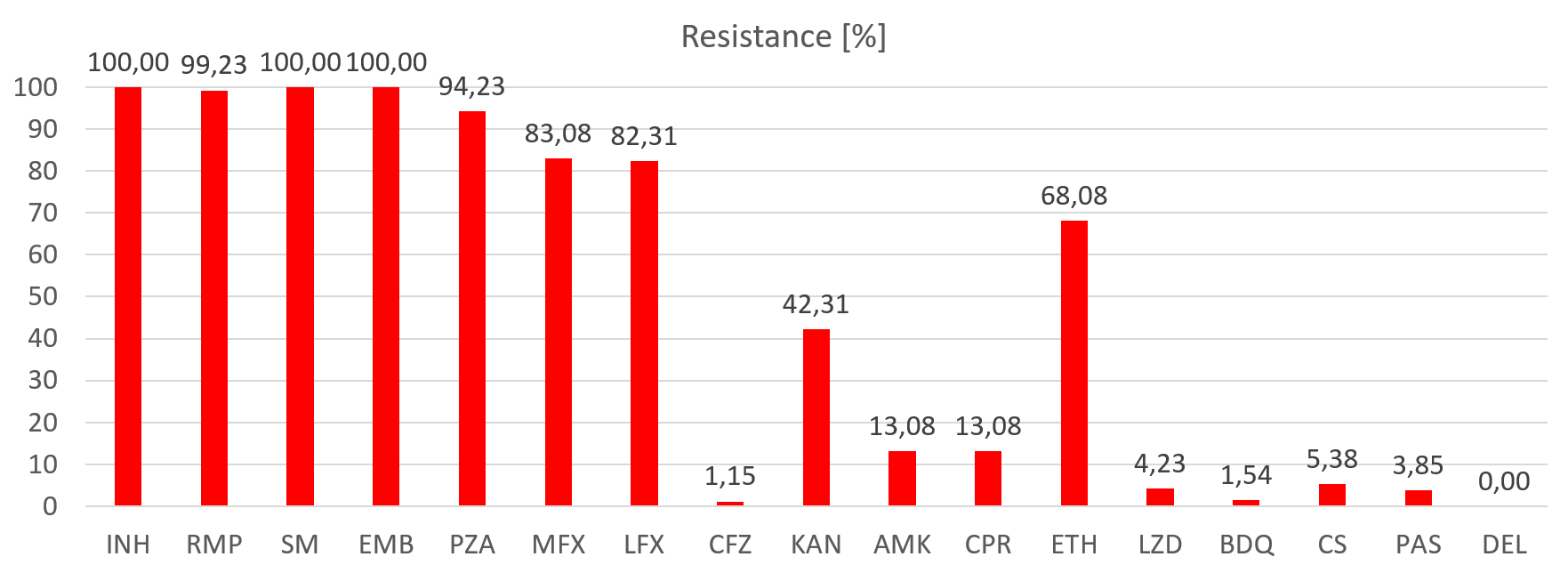


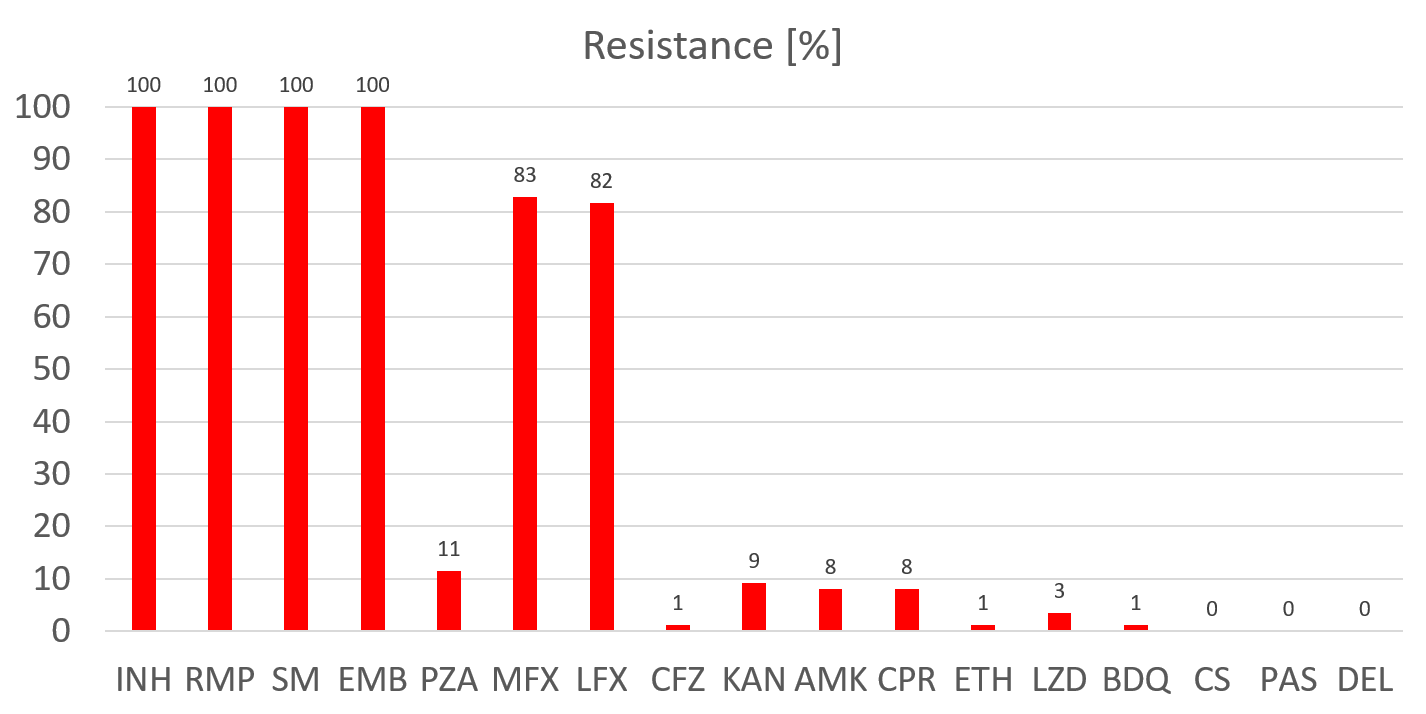
B


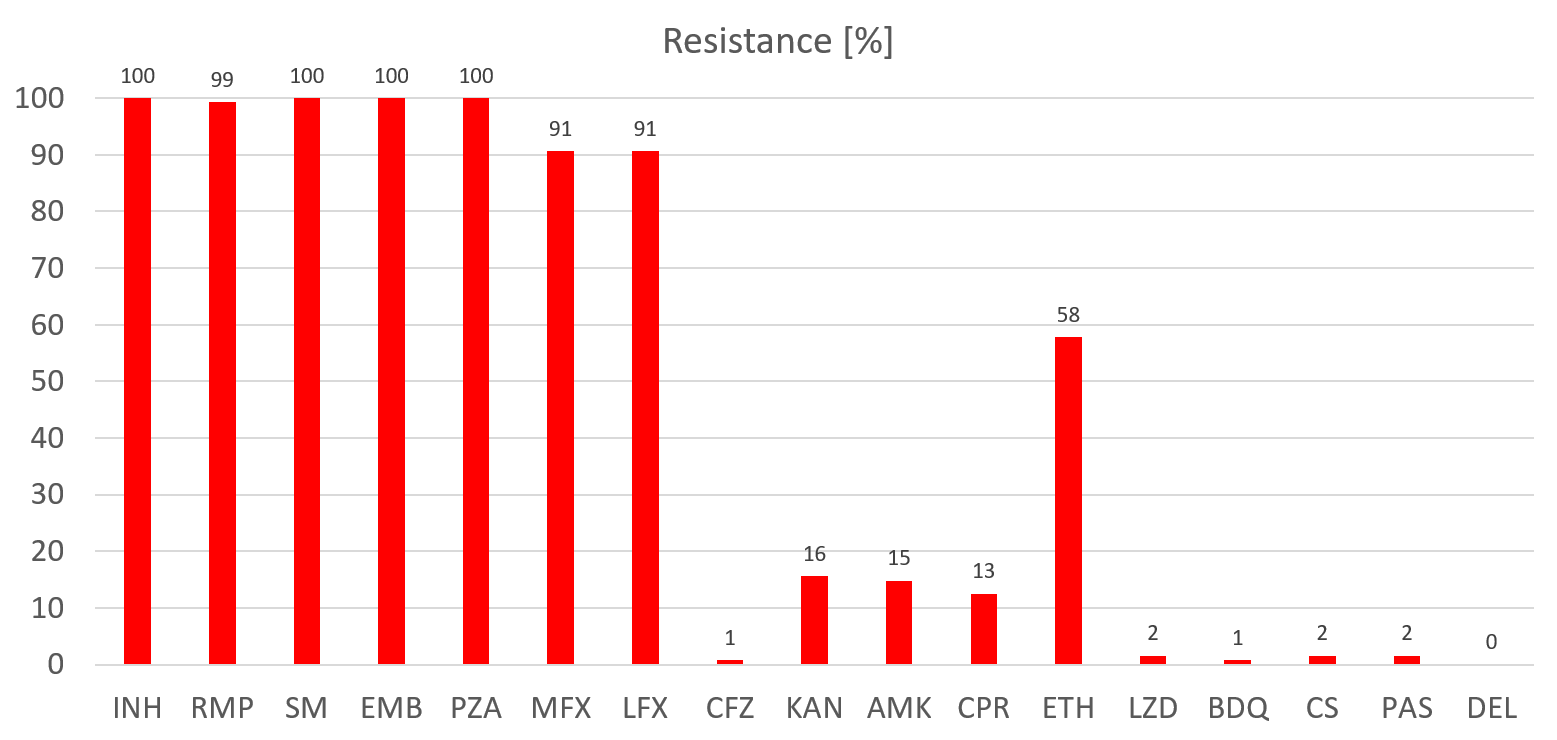
C

**Fig S4.** Barplot of resistance profiles of the strains belonging to allele cluster 1, 2 and 3. Resistance profiles in %. A) Resistance profile of allele Cluster 1, B. Resistance profile of allele Cluster 2, C. Resistance profile of allele Cluster 3

Abbreviations: INH - Isoniazid; RMP - Rifampicin; SM - Streptomycin; EMB - Ethambutol; PZA - Pyrazinamide; MFX - Moxifloxacin; LFX - Levofloxacin; CFZ - Clofazimine; KAN - Kanamycin; AMI - Amikacin; CPR - Capreomycin; ETH - Ethionamide; LZD - Linezolid; BDQ - Bedaquiline; CS - Cycloserine; PAS - para-aminosalycilic acid; DEL - Delamanid


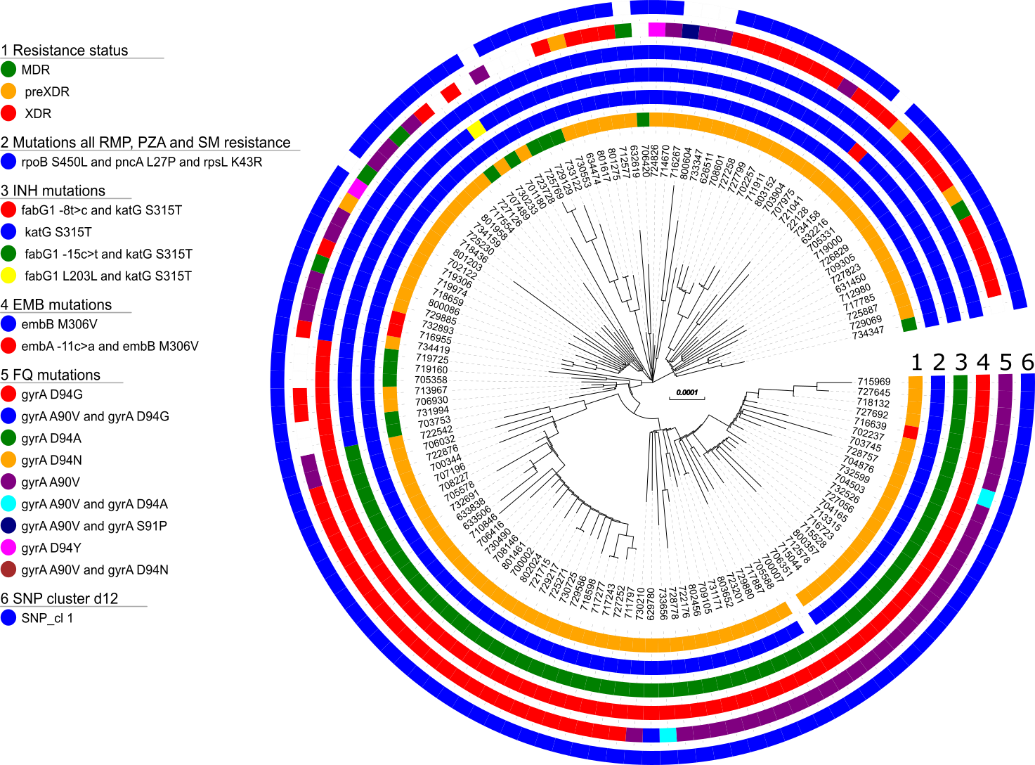
**A**

**
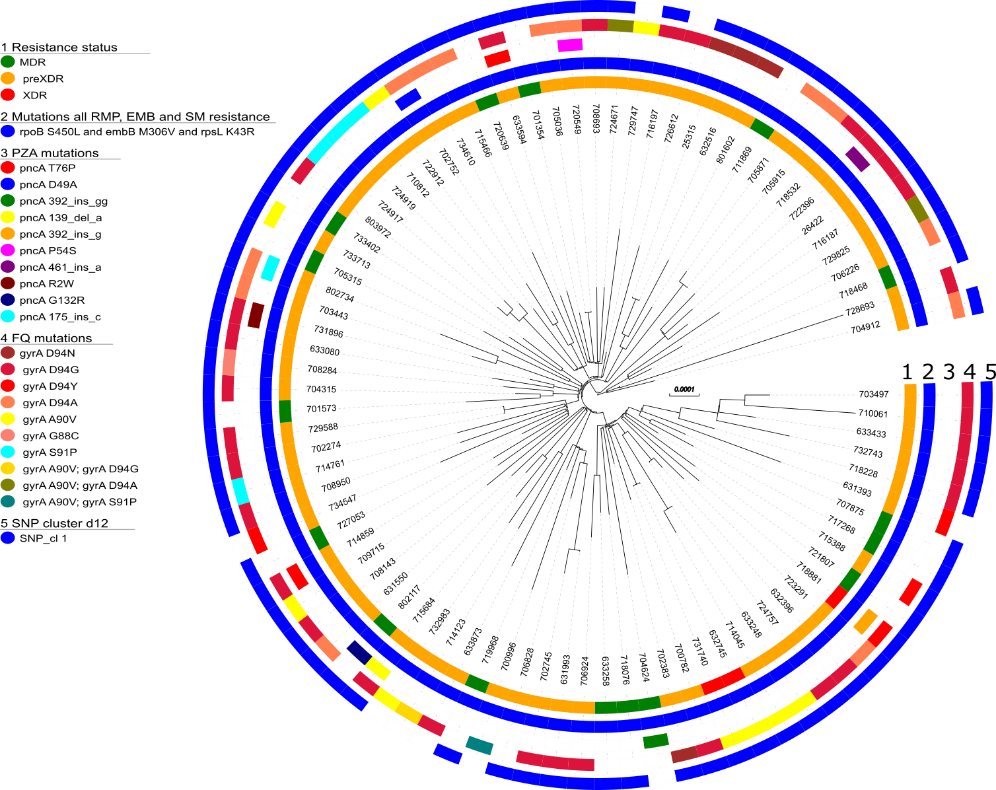
B**

**Fig S5.** Maximum likelihood (ML) phylogeny of strains belonging to cluster 2 and 3. Mutations related to respective drugs and resistance status are color coded on the annotation rings of the tree.
A. ML phylogenetic tree of the 127 MTBC strains from Cluster 2 phylogeny is based on the concatenated SNP sequence with 1 110 parsimony-informative and 386 singleton sites
B. ML phylogenetic tree of the 87 MTBC strains from Cluster 3, phylogeny is based on the concatenated SNP sequence with 122 parsimony-informative and 395 singleton sites
Abbreviations: INH - Isoniazid; EMB - Ethambutol; PZA - Pyrazinamide; FQ - Fluoroquinolones.

**
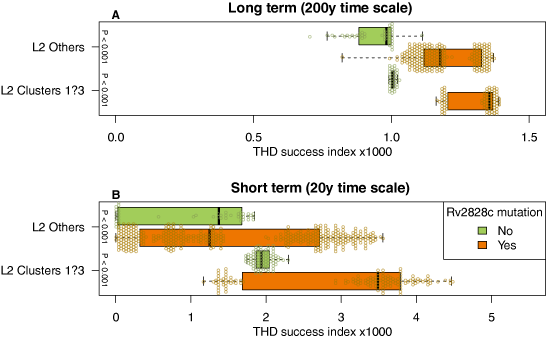
**

**Fig S6**. Rv2828c mutations correlate with increased epidemic success among MTBC lineage 2 isolates. Shown are boxplots and distribution of THD success indices in MTBC lineages 2 belonging or not to clusters 1-3. Among clusters 1-3 isolates, THD success indices were larger in isolates harboring Rv2828c mutations using both a long-term (A) and a short-term (B) analysis time scale. Among other L2 isolates, THD success indices were large in Rv2828c-positive isolates using a long-term timescale but not a short-term time-scale. This suggest that Rv2828c mutations were beneficial among L2 isolates in the long term, but only beneficial to cluster 1-3 isolates in the short term. P-values obtained from 2-sided Mann-Whitney U-test.
